# Correction: Reduced hot-electron energy-loss rate induced by finite-square confinement potential in GaN/AlN, GaAs/AlAs, and GaSb/InAs nanostructured materials

**DOI:** 10.1039/d6na90047j

**Published:** 2026-07-02

**Authors:** Ho Kim Dan, Pham Tuan Vinh, Le Phuong Long, Huynh Thi Phuong Thuy, Nguyen Dinh Hien

**Affiliations:** a Optical Materials Research Group, Science and Technology Advanced Institute, Van Lang University Ho Chi Minh City Vietnam hokimdan@vlu.edu.vn; b Faculty of Applied Technology, Van Lang School of Technology, Van Lang University Ho Chi Minh City Vietnam; c Division of Physics, School of Education, Dong Thap University Cao Lanh 870000 Vietnam; d Center of Scientific Research and Application, Lac Hong University No. 10 Huynh Van Nghe Str. Tran Bien Ward Dong Nai Province Vietnam; e Thu Dau Mot University Binh Duong Province Vietnam; f Institute of Research and Development, Duy Tan University Da Nang Vietnam; g School of Engineering & Technology, Duy Tan University Da Nang Vietnam nguyendinhhien2@duytan.edu.vn

## Abstract

Correction for ‘Reduced hot-electron energy-loss rate induced by finite-square confinement potential in GaN/AlN, GaAs/AlAs, and GaSb/InAs nanostructured materials’ by Ho Kim Dan *et al.*, *Nanoscale Adv.*, 2026, **8**, 3817–3829, https://doi.org/10.1039/d6na00063k.

The authors regret that the details of one of the affiliations (affiliation *b*) were incorrect in the original manuscript. The corrected list of affiliations is shown here.

The Royal Society of Chemistry apologises for these errors and any consequent inconvenience to authors and readers.

